# The MOCART (magnetic resonance observation of cartilage repair tissue) 2.0 Ankle Score

**DOI:** 10.1186/s13244-024-01696-7

**Published:** 2024-05-31

**Authors:** Markus M. Schreiner, Marcus Raudner, Carl S. Winalski, Vladimir Juras, Silke Aldrian, Alexander Kolb, Catharina Chiari, Reinhard Windhager, Siegfried Trattnig

**Affiliations:** 1 Department of Orthopedics and Trauma Surgery, Medical University of Vienna, Vienna, Austria; 2 High Field MR Center, Department of Biomedical Imaging and Image-Guided Therapy, Medical University of Vienna, Vienna, Austria; 3 Department of Diagnostic Radiology, Program of Advanced Musculoskeletal Imaging, Department of Biomedical Engineering, Lerner Research Institute, Cleveland Clinic, Cleveland, OH USA; 4 Austrian Cluster for Tissue Regeneration, Vienna, Austria; 5 CD Laboratory for Clinical Molecular MR Imaging, Vienna, Austria

**Keywords:** MRI, Cartilage, Ankle

## Abstract

**Objectives:**

The aim of this study was to introduce the MOCART 2.0 ankle score and evaluate its utility and reproducibility for the radiological assessment of cartilage repair tissue in the ankle joint.

**Methods:**

The MOCART 2.0 ankle score evaluates seven individual variables, including “volume fill of (osteo)chondral defect,” “Integration into adjacent cartilage and bone,” “surface of the repair tissue,” “signal intensity of the repair tissue,” “bony defect and bony overgrowth,” “presence of edema-like-marrow signal,” and “presence of subchondral cysts.” Overall, a MOCART 2.0 ankle score between 0 and 100 points may be reached. Two independent readers assessed the 3-T MRI examinations of 48 ankles, who had undergone cartilage repair of a talar cartilage defect using the new MOCART 2.0 ankle score. One of the readers performed two readings. Intra- and interrater reliability were assessed using intraclass correlation coefficients (ICCs) for the overall MOCART 2.0 ankle score.

**Results:**

Forty-eight ankles (mean age at surgery 30.2 ± 11.2 years) were evaluated. The overall interrater (ICC = 0.75; 95%CI 0.60–0.85), as well as the intrarater (ICC = 0.83; 95%CI 0.72–0.90) reliability of the MOCART 2.0 ankle score was good. For individual variables the interrater reliability ranged from a kappa value of 0.29 (95%CI 0.01–0.57) for “surface of the repair tissue” to 0.83 (95%CI 0.71–0.95) for “presence of subchondral cysts”.

**Conclusions:**

The newly introduced MOCART 2.0 ankle score, which encompasses the distinct anatomy of the ankle joint, demonstrates good intra- and interrater reliability.

**Critical relevance statement:**

The newly introduced MOCART 2.0 ankle score may facilitate the standardized assessment of cartilage repair in the ankle joint and allow an objective comparison of the morphological outcome between alternative treatment options and between different studies.

**Key Points:**

- This study introduces the MOCART 2.0 ankle score.
- The MOCART 2.0 ankle score demonstrated good intra- and interrater reliability.
- Standardized reporting may improve communication between radiologists and other physicians.

**Graphical Abstract:**

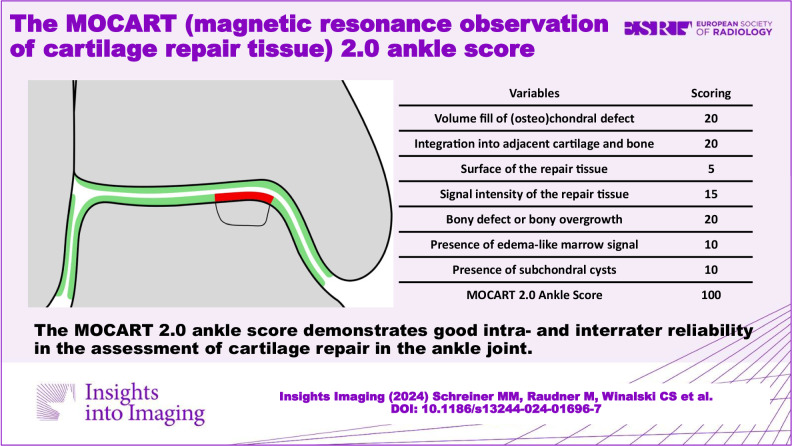

## Introduction

An increasing number of studies employ morphological and quantitative MRI in addition to clinical scores to assess outcome after cartilage repair in the ankle joint. The original MOCART score [1] and its recent update, the MOCART 2.0 knee score [2], have been developed to improve comparability and reproducibility between longitudinal follow-ups of individual patients as well as between different surgical cartilage repair techniques [3]. However, limited applicability and reproducibility of the original MOCART score have been demonstrated for the assessment of cartilage repair in the ankle joint [4]. While the updated MOCART 2.0 knee score demonstrated higher inter-and intrarater reproducibility when compared to the original MOCART score, it was developed specifically for application in the knee joint and does not meet the challenges, which are posed by the specific anatomy of the ankle joint and the higher frequency of osteochondral defects in this joint.

These osteochondral defects are most commonly located on the anterolateral or posteromedial talar dome [5]. Trauma has been described as the most common cause for lesions [6]. However, the trauma mechanism is thought to be different and both lesions exhibit a distinct morphology with medial lesions being deep and cup-shaped and lateral lesions being shallower and oval shaped [7].

Good reproducibility, however, is a prerequisite for a scoring system that should allow a meaningful comparison of the morphological outcome between the plethora of available treatment options for cartilage defects of the ankle joint, including debridement, curettage, and bone marrow stimulation [8], osteochondral autograft [9], (matrix-induced) autologous chondrocyte implantation [10], scaffold-based therapies [11], and the recently rediscovered technique of minced cartilage implantation [12].

Therefore, the aim of this study was to introduce the MOCART 2.0 ankle score, an adaption of the MOCART 2.0 knee score dedicated to the evaluation of cartilage repair tissue in the ankle and assess its intra- and interrater reproducibility.

## Materials and methods

This retrospective study was conducted according to the World Medical Association Declaration of Helsinki and was approved by the local institutional review board. Informed consent was waived. The study was conducted at the University Hospital Vienna of the Medical University of Vienna. We retrospectively identified all patients who underwent surgical cartilage repair for a talar cartilage lesion at our institution and who received at least one follow-up MR examination between 2007 and 2020. Six patients had to be excluded due to insufficient image quality due to metal artifacts or motion artifacts of the follow-up MR examination. Hence, 48 ankles of 47 patients were retrospectively included in the study.

### Variables of the MOCART 2.0 Ankle Score

**Fig. 1:**
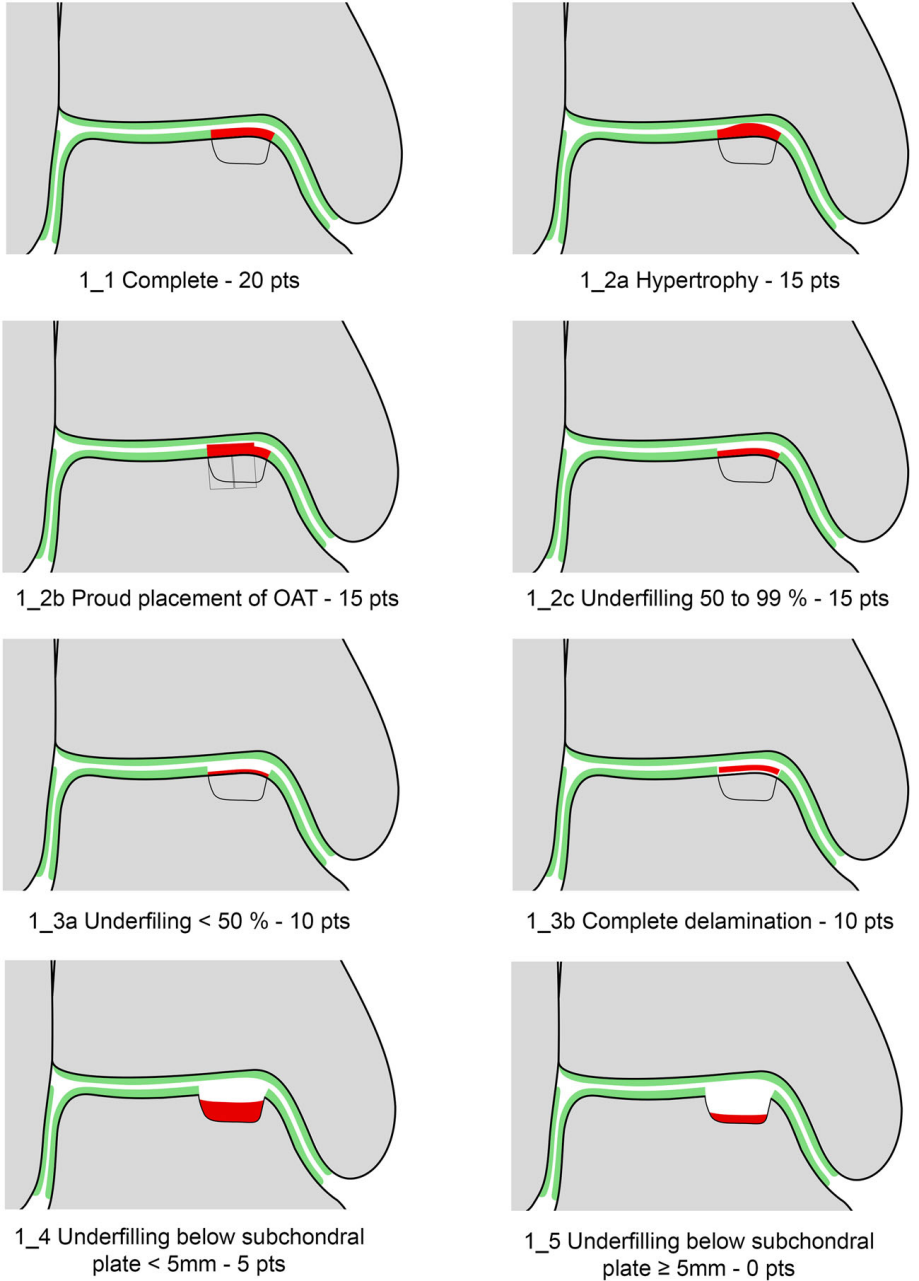
Volume fill of (osteo)chondral defect. Intact cartilage is depicted in green, repair tissue in red. The extent of the initial bony defect is indicated via a black line

**Fig. 2:**
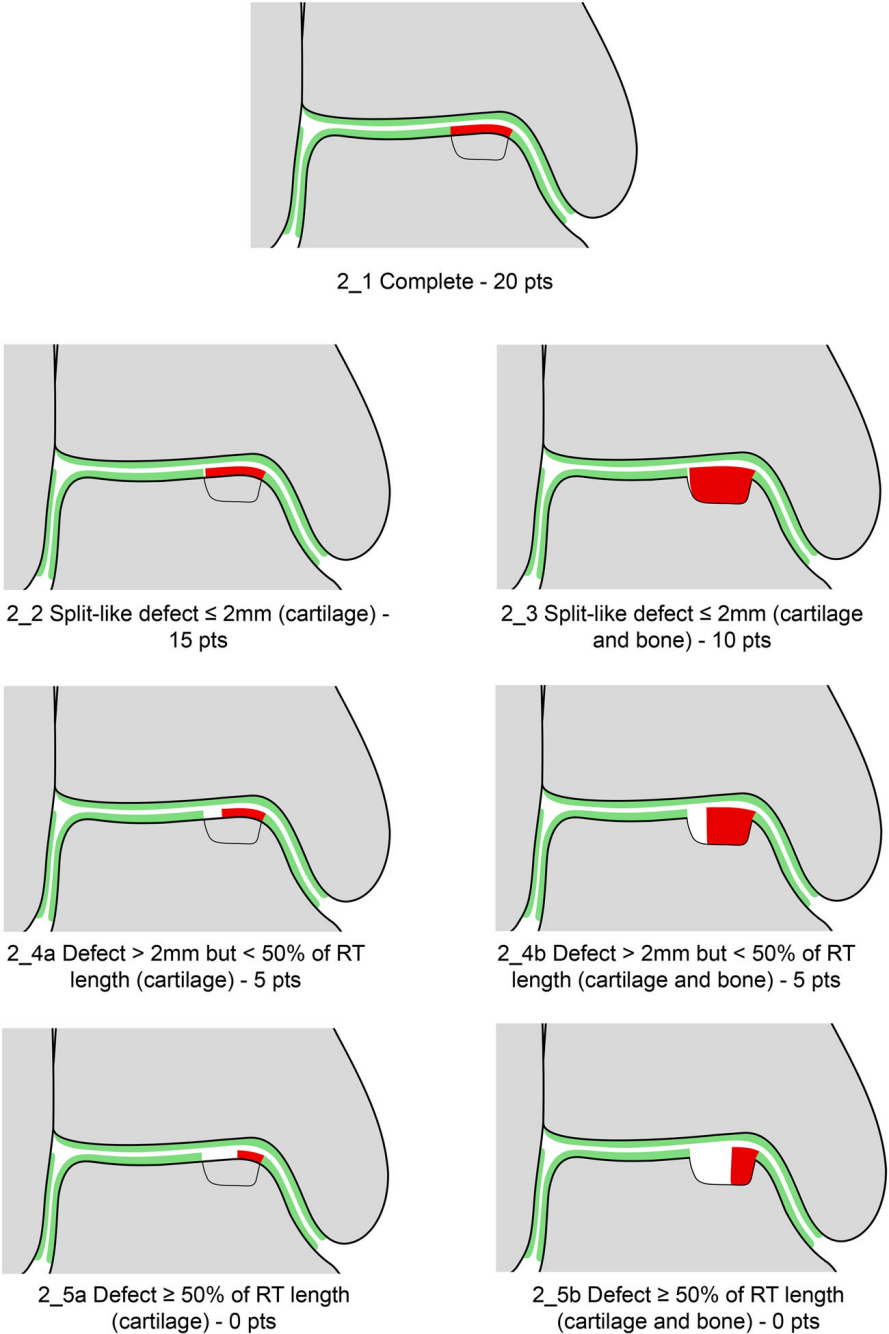
Integration into adjacent cartilage and bone. Intact cartilage depicted in green, repair tissue in red. The extent of the initial bony defect is indicated via a black line

**Fig. 3:**
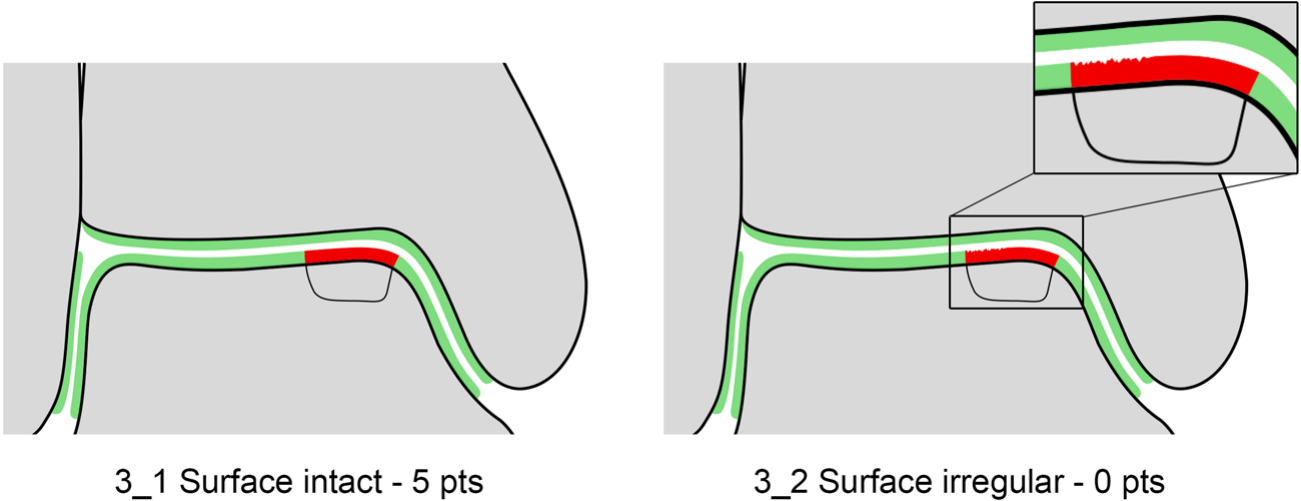
Surface of the repair tissue. Intact cartilage is depicted in green, repair tissue in red. The extent of the initial bony defect is indicated via a black line

**Fig. 4:**
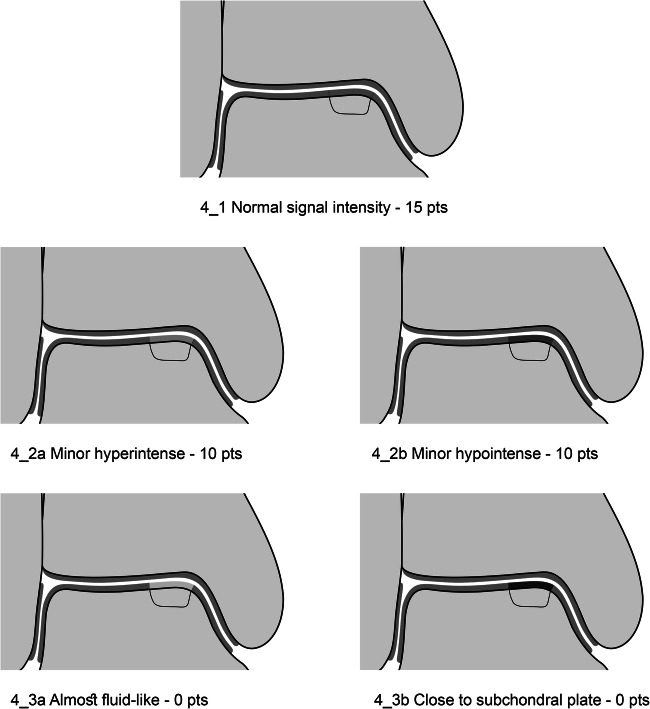
Signal intensity of the repair tissue. The extent of the initial bony defect is indicated via a black line

**Fig. 5:**
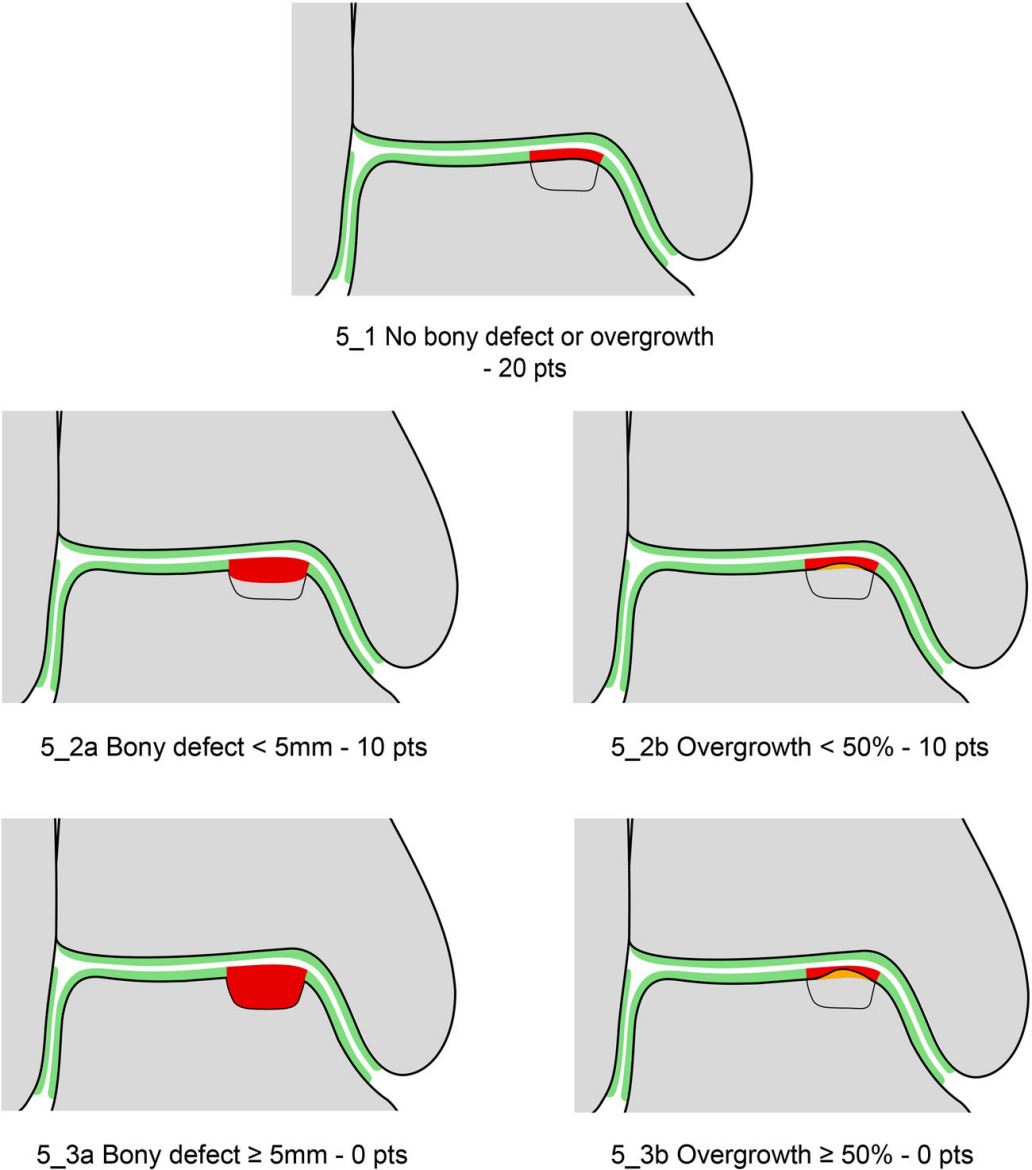
Bony defect or bony overgrowth. Intact cartilage is depicted in green, repair tissue in red, and overgrowth in orange. The extent of the initial bony defect is indicated via a black line

**Fig. 6:**
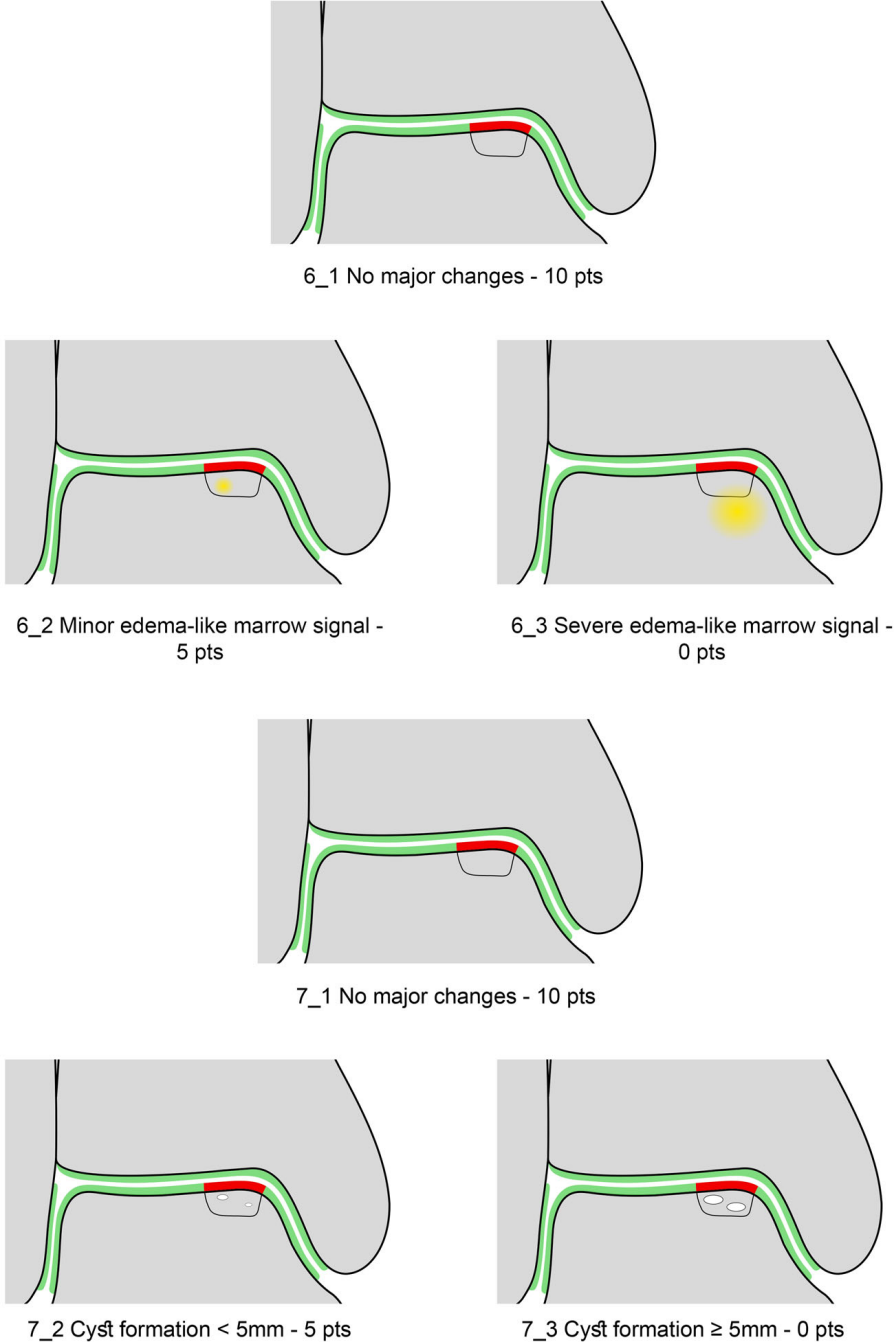
Presence of edema-like marrow signal in 6_1, 6_2 and 6_3; and the presence of subchondral cysts are evaluated separately as depicted in 7_1, 7_2, and 7_3. Intact cartilage is depicted in green, repair tissue in red, edema-like marrow signal in yellow. The extent of the initial bony defect is indicated via a black line

The seven variables of the recently introduced MOCART 2.0 knee score [2] were adapted to encompass the anatomy of the ankle joint. The variable “structure of the repair tissue” demonstrated inferior reproducibility upon evaluation of the first training data set and was discarded. Edema-like marrow signal and subchondral cysts, which are scored together in the MOCART 2.0 knee score as “subchondral changes,” were split into two variables in the MOCART 2.0 ankle score; this allowed a more comprehensive evaluation (Table 1). All variables are depicted on schematic drawings, as seen in Figs. 1–6.

**Table 1:** MOCART 2.0 ankle score: grading and point scale

**1**	**Volume fill of (osteo)chondral defect**		**Scoring**
	1	Complete filling: 100% filling of total defect volume (1_1)	20
	2	Hypertrophy (1_2a) or proud placement of AOT/allografts (1_2b) or 50–99% filling of total cartilage defect volume (1_2c) (or placement of osteochondral autografts/allografts below the cartilage surface with a step-off < 50% of neighboring cartilage thickness)	15
	3	< 50% filling of total cartilage defect volume (1_3a) (or placement of osteochondral autografts/allografts below the cartilage surface with a step-off > 50% of neighboring cartilage thickness) OR delamination in situ (1_3b)	10
	4	Defect filling below subchondral plate < 5 mm (1_4)	5
	5	Defect filling below subchondral plate ≥ 5 mm (1_5)	0
**2**	**Integration into adjacent cartilage and bone**		
	1	Complete integration (2_1)	20
	2	Split-like defect at repair tissue—native cartilage interface ≤ 2 mm (2_2)	15
	3	Split-like defect at repair tissue—native cartilage AND bone interface ≤ 2 mm (2_3)	10
	4	Full-thickness integration defect at repair tissue - native cartilage (2_4a) (AND bone interface (2_4b)) > 2 mm, but < 50% of repair tissue length	5
	5	Full-thickness integration defect at repair tissue and native cartilage (2_5a) (AND bone interface (2_5b)) ≥ 50% of repair tissue length	0
**3**	**Surface of the repair tissue**		
	1	Surface intact (3_1)	5
	3	Surface irregular (3_2)	0
**4**	**Signal intensity of the repair tissue**		
	1	Normal (4_1)	15
	2	Minor abnormal—minor hyperintense (4_2a) OR minor hypointense (4_2b)	10
	3	Severely abnormal—almost fluid like (4_3a) OR close to subchondral plate signal (4_3b)	0
**5**	**Bony defect or bony overgrowth**		
	1	No bony defect or bony overgrowth (5_1)	20
	2	Bony defect < 5 mm (5_2a) OR overgrowth < 50% of adjacent cartilage thickness (5_2b)	10
	3	Bony defect ≥ 5 mm (5_3a) OR overgrowth ≥ 50% of adjacent cartilage thickness (5_3b)	0
**6**	**Presence of edema-like marrow signal**		
	1	No edema-like marrow signal (6_1)	10
	2	Minor edema-like marrow signal: < 10 mm (6_2)	5
	3	Severe edema-like marrow signal: ≥ 10 mm (6_3)	0
**7**	**Presence of subchondral cysts**		
	1	No subchondral cysts (7_1)	10
	2	Subchondral cyst/cyst formation < 5 mm in longest diameter (7_2)	5
	3	Subchondral cyst/cyst formation ≥ 5 mm in longest diameter (7_3)	0

#### Volume fill of (osteo)chondral defect

Despite the improved signal-to-noise ratio that can be achieved with recent developments in MR imaging, the thin and highly congruent cartilage layers of the ankle joint pose a greater challenge for assessment of the volume of cartilage defect filling than for the knee. Hence the “volume fill of the (osteo)chondral defect” was adapted to 50% increments for the ankle joint. The volume of defect filling must be assessed in relation to the adjacent native reference cartilage and must be described as a percentage of the hypothetical volume of intact cartilage that covers the defect. It has been previously reported that ankle cartilage thickness is relatively homogenous throughout the joint with similar thickness reported for tibial and talar cartilage [13]. Thus, if the articular surfaces of the tibia and talus cannot be separately identified on MR images, the thickness of the native cartilage should be assumed to be half of the distance between subchondral bone plates. Since there is a high percentage of osteochondral lesions in the ankle joint, greater attention was paid to the subchondral bone. Therefore, the volume fill of the defect variable for the ankle also includes an assessment of the volume fill or restoration of the subchondral bone in case of osteochondral lesions. The volume of filling is considered complete (100%) and scored with 20 points when the repair tissue articular surface is flush with the surrounding reference cartilage with a repair tissue volume equivalent to the hypothetical volume of healthy cartilage that would cover the defect (Fig. 1_1). A repair with incomplete cartilage defect filling compared to adjacent native regions is classified as underfilled and can be “minor underfilling” (50–99% filled, Fig. 1_2c), which is scored with 15 points, or “severe underfilling” (< 50%, Fig. 1_3a), which is scored with 10 points. Complete chondro-osseous delamination in situ (Fig. 1_3b) receives the same score as severe underfilling as it bears the risk of cartilage fragment dislocation and subchondral bone exposure. On MR imaging, chondro-osseous delamination is characterized by a fissure-like, fluid-like interface between the repair tissue and subchondral bone. Although delamination may subsequently resolve in the early postoperative period, once maturation of cartilage is complete, the finding indicates that further repair to bone incorporation is unlikely. If the defect extends into the subchondral bone, the depth of the bony defect has to be measured. A bony defect, which is not filled with repair tissue and is less than 5 mm deep is scored with five points (Fig. 1_4), a bony defect equal to or greater 5 mm and not filled with repair tissue is scored with 0 points (Fig. 1_5).

For the knee joint, Kreuz et al [14] found that hypertrophic repair tissue <150% does not negatively impact clinical outcome; however, this is not true for the ankle joint. Hence, hypertrophy of any degree (Fig. 1_2a) is scored the same as minor underfilling (50–99%) and is scored with 15 points. Evaluation in at least two different pulse sequences and planes is essential since graft hypertrophy can be easily missed or underestimated, especially on fat-suppressed images. In case of incongruity of the surface between an osteochondral graft and host cartilage after osteochondral transfer, a so-called graft height mismatch, this surface incongruity should be scored in the same way as a corresponding amount of hypertrophy or underfilling. It has been previously demonstrated that graft height mismatch may have significant impact on the pressures forces on the graft itself or the opposite facet of the talus alike [15]. If the cartilage surface of the osteochondral graft is proud (1_2b) it is scored with 15 points. If the cartilage surface of the osteochondral graft is below the surface of neighboring cartilage, it is scored in the same way as a corresponding degree of underfilling.

### Integration into adjacent cartilage and bone

This variable evolved from the original knee MOCART variable “integration with border zone”. It serves as a measure of the integration of the cartilage repair tissue into the neighboring native cartilage by evaluating the interface between these tissues. Due to the high prevalence of osteochondral lesions an extension to the bony part of the lesion was introduced. Integration is classified as complete (Fig. 2_1) in cases of an indiscernible interface between the repair tissue and the adjacent cartilage and bone. In case of a hyperintense (fluid-like) demarcation or separation between the repair tissue and the adjacent cartilage, the width of this defect must be determined. If the fluid-like split-like defect is ≤ 2 mm, additional extension of the defect to the subchondral bone has to be assessed. If the split-like defect ≤ 2 mm is restricted to the cartilage layer (Fig. 2_2), 15 points are allocated. If the split-like defect ≤ 2 mm extends to the subchondral bone (Fig. 2_3), 10 points are scored. Defects > 2 mm but < 50% of the repair tissue length (Fig. 2_4a and Fig. 2_4b) and defects ≥ 50% of the repair tissue length (Fig. 2_5a and Fig. 2_5b) are scored regardless of the extension to subchondral bone with 5 and 0 points, respectively.

#### Surface of the repair tissue

It is important to assess the surface of the repair tissue independently of the volume of cartilage defect filling. The surface must be evaluated with respect to the presence of irregularities, regardless of perfect filling, hypertrophy, or underfilling. To be able to visualize fine fibrillations and fissures on the surface of the repair tissue, high-resolution MR imaging protocols are essential. Since the ankle is a highly congruent joint, it may be difficult to visualize the separation between the tibial and talar cartilage. Hence, this variable was simplified to a dichotomous variable with the surface of the repair tissue being either classified as “intact” in the case of a preserved, congruent, or unidentifiable articular surface (Fig. 3_1) or “irregular” in the case of visible surface irregularities (Fig. 3_2). Irregularities of the articular surface may range from minor fibrillations to fissures and ulcerations.

#### Signal intensity of the repair tissue

As with the MOCART 2.0 knee score, signal intensity of the repair tissue should be evaluated on all available fat-saturated as well as non-fat-saturated proton density-weighted turbo spin echo (PDw-TSE) sequences, which offer a higher sensitivity for the intrachondral structure of cartilage than T2-weighted acquisition. Similar to the MOCART 2.0 knee score, repair tissue signal alterations are rated hyper- or hypointense with possible scorings of “normal” (isointense to adjacent native cartilage) (Fig. 4_1), “minor abnormal” (Fig. 4_2a: “minor hyperintensity” and Fig. 4_2b: “minor hypointensity”), and “severely abnormal” (Fig. 4_3a: “almost fluid-like signal” and Fig. 4_3b: “almost subchondral plate signal”). An abnormal signal intensity on one sequence is sufficient for grading as abnormal; however, the abnormality should be present in more than one slice or imaging plane to avoid inaccurate interpretation due to partial volume averaging or other artifacts. In addition, the most severe abnormality should be scored, e.g., if the repair tissue shows minor hypointensity and major hypointensity in different regions, it should receive 0 points (major hypointensity). The influence of the magic angle effect [16] must be considered when evaluating the repair tissue signal intensity. Should the repair tissue be located at the shoulder of the talus at an angle close to 55° to the direction of the magnet bore, B0, the intensity should be evaluated in reference to healthy cartilage at a similar angulation to avoid false interpretation. While hyperintensity of the repair tissue may represent a higher water content and disorganization of the collagen fiber network, hypointensity of the repair tissue on the same sequence may result from fibrous tissue formation. Overall, this variable may be an indicator of tissue maturation during the early post-operative phase, usually up to one year [17].

#### Bony defect, bony overgrowth, or mismatch of the subchondral bone plate

As previously mentioned, due to the high percentage of osteochondral lesions of all cartilage defects of the talus, cartilage repair in the ankle joint frequently must restore the entire osteochondral unit. Thus, subchondral bone assessment is of particular importance for the MOCART 2.0 ankle score. A perfect outcome with intact, congruent subchondral bone and no presence of intrachondral osteophytes, would be scored “no bony defect or bony overgrowth” (Fig. 5_1). Bony defects after osteochondral allograft or autograft transfer should be subcategorized according to depth. Since the cartilage layer of the talus is significantly thinner than the cartilage in the knee joint, the depth of the bony defect is not evaluated in comparison to the thickness of the neighboring intact cartilage, but rather in comparison to a cutoff of 5 mm (Fig. 5_2a and 5_3a). Bony overgrowth or intrachondral osteophytes should be subcategorized as bony overgrowth < 50% (Fig. 5_2b) and ≥ 50% (Fig. 5_3b) of the thickness of the adjacent native cartilage. If the articular surfaces of the tibia and talus cannot be separately identified on MR images, the thickness of the native cartilage should be assumed to be half of the distance between subchondral bone plates. The extent of bony overgrowth should always be assessed using the adjacent native cartilage as a reference, especially in case of an underfilling of the defect, in which the repair tissue thickness used as a reference might produce a false positive result.

#### Presence of edema-like marrow signal

The assessment of edema-like marrow signal and subchondral cysts, which was combined into the variable “subchondral changes” in the MOCART 2.0 knee score, was split into two variables to allow for a separate, more comprehensive evaluation. Edema-like marrow signal is best appreciated as an ill-defined hyperintense area of the subchondral bone marrow on fluid-sensitive sequences or an area of ill-defined intermediate or low signal intensity on a T1 weighted sequence when compared to normal subchondral bone [18, 19]. When the bone marrow beneath the subchondral bone lamina appears normal, the variable “presence of edema-like marrow signal” is rated “No edema-like marrow signal” (Fig. 6_1) with 10 points. If there is an edema-like marrow signal present it can be subdivided into minor, with a maximum diameter less than 10 mm (Fig. 6_2) and scored with 5 points, or severe, with a maximum diameter of 10 mm or greater (Fig. 6_3) scored with 0 points.

#### Presence of subchondral cysts

This variable was introduced to allow for a separate evaluation of the presence of subchondral cysts. If there are no subchondral cysts present, this variable is scored with 10 points. For subchondral cysts with an individual or combined diameter < 5 mm (i.e., multiple small cysts have a combined diameter < 5 mm), 5 points are scored. For subchondral cysts with an individual or combined diameter ≥ 5 mm (i.e., multiple small cysts have a combined diameter ≥ 5 mm), zero points are allocated in this variable.

### Magnetic resonance imaging

Imaging was performed on different 3-T MR systems (MAGNETOM Tim Trio, MAGNETOM Verio, MAGNETOM Prisma, Siemens Healthineers) and was part of the clinical routine follow-up. Therefore, the imaging protocols and sequence parameters differed slightly between patients. An exemplary routine MRI protocol that was used in the study is given in Table 2. The protocol included a three-dimensional localizer, followed by a sagittal fat-saturated proton-density-weighted turbo spin-echo (sag PDw TSE) sequence, a sagittal T1-weighted (T1w) TSE, and a coronal fat-saturated PDw TSE sequence.

**Table 2:** Exemplary MRI protocol that fulfills the recommended requirements in terms of sequences and resolution for adequate assessment of the MOCART 2.0 ankle score at 3 T

	Example parameters for a 3-T protocol		
	Sagittal PDw TSE fs	Sagittal T1w TSE	Coronal PDw TSE fs
**TE [ms]**	25	9.7	25
**TR [ms]**	2100	788	2100
**Flip angle**	160	145	160
**Fat suppression**	Yes	None	Yes
**FOV (mm)**	100	150	100
**RFOV (%)**	100	100	100
**Acq. matrix**	320	384	320
**Slices**	15	26	15
**Slice thickness**	3	3	3
**Interslice gap (%)**	20	10	50
**Slice orientation**	sagittal	sagittal	coronal
**Acquisition time [TA]**	06:32	03:06	06:32

*Fs* Fat-saturated, *PDw* Proton-density-weighted, *TSE* Turbo spin-echo

### Image analysis

Image analysis was performed on a picture archiving and communication system (PACS) workstation (IMPAX EE R20, Agfa Healthcare N.V., Mortsel, Belgium) by two independent readers: one board-certified radiologist (reader 1) and one board-certified orthopedic surgeon (reader 2) each with 8 years of experience in musculoskeletal MR imaging (reader 1 and reader 2). Imaging studies were assessed in random order, and all readers were blinded to all patient details. An eight-week interval was maintained between the first and the second reading of reader 1.

### Statistical analysis

All statistical calculations were performed using IBM SPSS Statistics for Windows version 25 (IBM, Armonk, NY, USA). Metric data are described using mean ± standard deviation. Linear weighted kappa statistics and their 95% confidence intervals were calculated as an index for inter- and intrarater reliability of each ordinal scoring domain of the MOCART 2.0 ankle osteochondral score. Weighted kappa statistics were interpreted according to the criteria of Landis and Koch[20]. A kappa value ≤ 0.20 indicated poor agreement, 0.21–0.40 indicated fair agreement, 0.41–0.60 indicated moderate agreement, 0.61–0.80 indicated substantial agreement, and a kappa value of ≥ 0.81 indicated almost perfect agreement. Intraclass correlation coefficients (ICCs) (two-way mixed, absolute agreement, single measures) and their 95% confidence intervals (CI) were calculated as an index of intra-rater and inter-rater reliability for the overall MOCART 2.0 ankle osteochondral score. ICCs were interpreted according to Koo and Li [21], an ICC of less than 0.5 indicated poor agreement, 0.50–0.75 moderate agreement, 0.75–0.90 good agreement, and an ICC of above 0.90 excellent agreement. For the categorical variables, absolute agreement was calculated.

## Results

### Patients

A total of 48 ankles of 47 patients (24 female, 23 male), who underwent surgical cartilage repair for a talar cartilage lesion and who received follow-up MR examinations, were retrospectively included in the study. Twenty-two ankles were treated with microfracture, 17 ankles were treated with autologous chondrocyte transplantation, five of which received additional autologous spongiosaplasty, four ankles underwent cartilage repair with Maioregen® (Fin-ceramica), three ankles were treated with mosaicplasty and two ankles were treated with autologous spongiosaplasty and Hyalofast®. The following matrices were used in our study population for autologous chondrocyte implantation: Hyalograft C® (Anika Therapeutics), Carticel® (Vericel), Novocart 3D® (Braun Medical) and CaReS® (Arthro-Kinetics). Mean age at surgery was 30.2 ± 11.2 years. The mean follow-up time between index surgery and MRI was 4.0 ± 3.5 years, ranging from two months to 13.8 years.

### Inter- and intrarater reliability of the MOCART 2.0 ankle score

Both the inter- and interrater reliability of the overall MOCART 2.0 ankle score was good, according to Koo and Li, with an ICC of 0.832 (95%CI 0.719–0.903) and 0.754 (95%CI 0.601–0.854), respectively. For individual variables, the interrater reliability ranged from a kappa value of 0.290 (95%CI 0.014–0.567) for “surface of the repair tissue” to 0.833 (95%CI 0.714–0.951) for “presence of subchondral cysts”. Similarly, intrarater reliability ranged from a weighted kappa value of 0.492 (95%CI 0.233–0.751) for the variable “surface of the repair tissue” to 0.784 (95%CI 0.652–0.916) for the variable “subchondral cysts” (Table 3).

**Table 3:** Interrater and intrarater reliability of the MOCART 2.0 ankle score given as weighted kappa statistics for individual variables and two-way mixed absolute agreement ICC and their 95% confidence intervals (95%CI) for the resulting total MOCART 2.0 ankle score

Interrater and intrarater reliability of the MOCART 2.0 ankle score					
Variables MOCART 2.0 ankle score	Interrater reliability		Variables MOCART 2.0 ankle score	Intrarater reliability	
	ICC	95%CI		ICC	95%CI
Overall	0.754	0.601–0.854	Overall	0.832	0.719–0.903
	**Kappa**	**95%CI**		**Kappa**	**95%CI**
**Volume fill**	0.525	0.310–0.739	**Volume fill**	0.688	0.494–0.882
**Integration**	0.585	0.259–0.912	**Integration**	0.748	0.537–0.959
**Surface**	0.290	0.014–0.567	**Surface**	0.492	0.233–0.751
**Signal intensity**	0.449	0.234–0.665	**Signal intensity**	0.635	0.444–0.827
**Bony defect/overgrowth**	0.696	0.474–0.918	**Bony defect/overgrowth**	0.733	0.532–0.934
**Edema-like marrow signal**	0.686	0.525–0.848	**Edema-like marrow signal**	0.704	0.540–0.867
**Cysts**	0.833	0.714–0.951	**Cysts**	0.784	0.652–0.916

## Discussion

The aim of this study was to introduce the MOCART 2.0 ankle score, an adaption of the MOCART 2.0 knee score, which is dedicated to the noninvasive morphological assessment of cartilage repair tissue in the ankle joint and encompasses the distinct anatomy, as well as pathophysiology of cartilage defects and repair in the ankle joint.

The main finding of the study was that the interrater and intrarater reproducibility was good. As expected, both the interrater as well as the intrarater reproducibility differed between individual variables. Both the interrater as well as the intrarater reproducibility were lowest for the variable “surface of the repair tissue.” This was already true for the MOCART 2.0 knee score [3], albeit at higher levels. This difference can be explained by the highly congruent cartilage layers of the tibia and the talus in the ankle joint, which render accurate discrimination, as well as decision on the state of the surface particularly difficult. However, considering the importance of the integrity of the surface of the repair tissue, it was decided to keep this variable in the scoring system.

In comparison to the knee joint, osteochondral lesions make up a higher percentage of cartilage lesions in the ankle joint. Hence, restoration of the subchondral bone is a particular focus of the MOCART 2.0 ankle score, and the variable volume fill was extended to the subchondral bone. Furthermore, changes of the subchondral bone, including edema-like marrow signal, as well as subchondral cysts are frequently observed and may correlate with clinical outcome [22, 23]. Given the importance of these pathologies, they are scored as two separate variables to allow more comprehensive scoring.

This study has certain limitations that need to be addressed. First, imaging studies were conducted on different scanners and vendors. Hence, sequence parameters differed slightly between patients, which introduces some variability. However, image quality was checked before inclusion, and six patients were excluded due to inadequate image quality due to movement artifacts or lack of specific sequences, which would have rendered reproducible assessment impossible.

Furthermore, a comparison of the reproducibility of the MOCART 2.0 ankle score between different repair techniques would have been interesting. However, the patient numbers for individual repair techniques were too small to perform such a subgroup analysis.

The MOCART 2.0 ankle score demonstrated good applicability and reproducibility for the assessment of cartilage repair in the ankle joint. However, additional studies comparing the reproducibility of the MOCART 2.0 ankle score for the assessment of different cartilage repair techniques, as well as studies that include clinical data as well are warranted.

## Data Availability

The data underlying this article cannot be shared publicly for the privacy of individuals that participated in the study. The data underlying this article will be shared on reasonable request to the corresponding author.

## Abbreviations

CI: Confidence interval
Fs: Fat saturated
ICC: Intraclass correlation coefficient
MOCART: Magnetic Resonance Observation of Cartilage Repair Tissue
MRI: Magnetic resonance imaging
PDW: Proton density-weighted
T1w: T1-weighted
TSE: Turbo spin echo
